# Image analysis of cutaneous melanoma histology: a systematic review and meta-analysis

**DOI:** 10.1038/s41598-023-31526-7

**Published:** 2023-03-23

**Authors:** Emily L. Clarke, Ryckie G. Wade, Derek Magee, Julia Newton-Bishop, Darren Treanor

**Affiliations:** 1 Department of Histopathology, Leeds Teaching Hospitals NHS Trust, Leeds, UK; 2 Division of Pathology and Data Analytics, Leeds Institute of Cancer and Pathology, University of Leeds, Beckett Street, Leeds, LS9 7TF UK; 3 Leeds Institute for Medical Research, University of Leeds, Leeds, UK; 4 School of Computing, University of Leeds, Leeds, UK; 5 Department of Clinical Pathology, and Department of Clinical and Experimental Medicine, Linköping University, Linköping, Sweden; 6 Center for Medical Image Science and Visualization (CMIV), Linköping University, Linköping, Sweden

**Keywords:** Biomarkers, Medical research, Diagnosis, Prognosis

## Abstract

The current subjective histopathological assessment of cutaneous melanoma is challenging. The application of image analysis algorithms to histological images may facilitate improvements in workflow and prognostication. To date, several individual algorithms applied to melanoma histological images have been reported with variations in approach and reported accuracies. Histological digital images can be created using a camera mounted on a light microscope, or through whole slide image (WSI) generation using a whole slide scanner. Before any such tool could be integrated into clinical workflow, the accuracy of the technology should be carefully evaluated and summarised. Therefore, the objective of this review was to evaluate the accuracy of existing image analysis algorithms applied to digital histological images of cutaneous melanoma. Database searching of PubMed and Embase from inception to 11th March 2022 was conducted alongside citation checking and examining reports from organisations. All studies reporting accuracy of any image analysis applied to histological images of cutaneous melanoma, were included. The reference standard was any histological assessment of haematoxylin and eosin-stained slides and/or immunohistochemical staining. Citations were independently deduplicated and screened by two review authors and disagreements were resolved through discussion. The data was extracted concerning study demographics; type of image analysis; type of reference standard; conditions included and test statistics to construct 2 × 2 tables. Data was extracted in accordance with our protocol and the Preferred Reporting Items for Systematic Reviews and Meta-Analyses-Diagnostic Test Accuracy (PRISMA-DTA) Statement. A bivariate random-effects meta-analysis was used to estimate summary sensitivities and specificities with 95% confidence intervals (CI). Assessment of methodological quality was conducted using a tailored version of the Quality Assessment of Diagnostic Accuracy Studies (QUADAS-2) tool. The primary outcome was the pooled sensitivity and specificity of image analysis applied to cutaneous melanoma histological images. Sixteen studies were included in the systematic review, representing 4,888 specimens. Six studies were included in the meta-analysis. The mean sensitivity and specificity of automated image analysis algorithms applied to melanoma histological images was 90% (CI 82%, 95%) and 92% (CI 79%, 97%), respectively. Based on limited and heterogeneous data, image analysis appears to offer high accuracy when applied to histological images of cutaneous melanoma. However, given the early exploratory nature of these studies, further development work is necessary to improve their performance.

## Introduction

**Figure 1:**
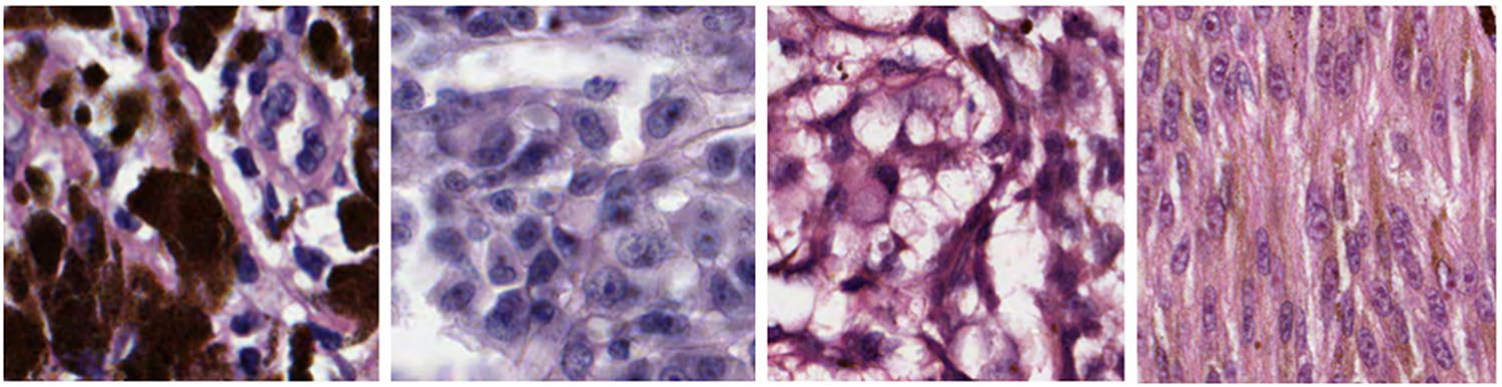
An example of the range of histopathological appearances of melanoma. The first image on the far left shows a tumour in which the tumour cells are obscured by large amounts of melanin pigment; the second image from the left shows a more conventional melanoma without pigment; the third image from the left shows a balloon cell variant of melanoma; the image on the right is an example of a spindle cell melanoma. This is an original image created by the authors using Medical Image Manager, HeteroGenius Limited, UK.

Despite advances in therapy, the 5-year survival of patients with metastatic melanoma is still less than 30%^1^. Moreover, the incidence of melanoma is predicted to rise by 7% from 2014 to 2035^2^. Diagnosis of melanoma depends upon a histopathologist’s interpretation of the tissue at a cellular level, with subjective thresholds for morphological features. Histopathological interpretation can be challenging, resulting in high levels of interobserver variation^3^, which may in part be due to the wide range of histological appearances (see Fig. 1). Consequently, up to 17% of diagnoses are reclassified as false positives or false negatives when reviewed by a specialist panel of pathologists^3^.

The current prognostic biomarkers based on histological features are contained within the current staging system (American Joint Committee on Cancer, AJCC)^4^, with maximum tumour depth (Breslow thickness) remaining the most important predictor of survival for over 50 years^5^. Other prognostic biomarkers contained within the AJCC staging include ulceration, mitoses, lymph node involvement and metastatic disease detected in viscera. However, the staging system explains an insufficient proportion of the variance in survival^6^ with some thin tumours unexpectedly causing metastatic disease.

Given that there is currently a staffing shortage in pathology services globally, with only 3% of pathology departments reporting being fully staffed in the United Kingdom (UK)^7^, there is a clear need for tools that aid pathologists and improve workflow. It is important that any new prognostic biomarkers not demand additional work of histopathologists.

**Figure 2:**
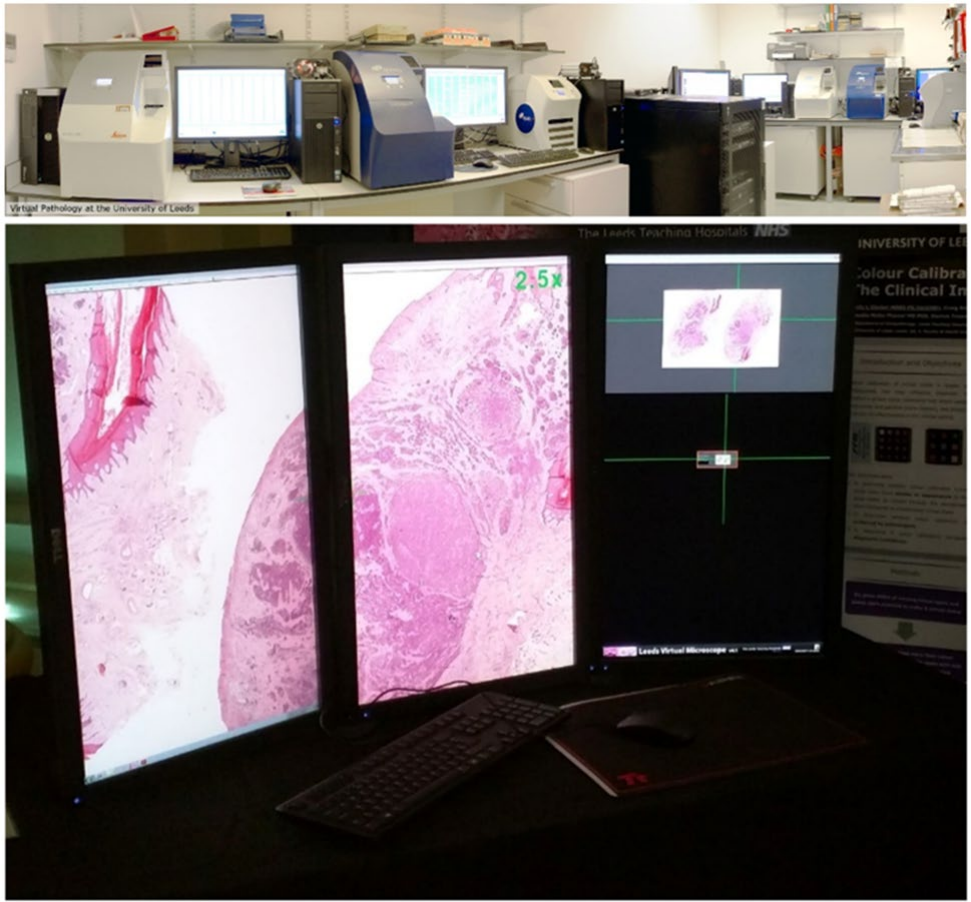
Glass slides are scanned using a slide scanner (photo above, credit: Mike Hale, University of Leeds) to create a whole slide image (image below) which can be viewed on a computer display. This digitisation of whole slide images has permitted the application of image analysis algorithms.

Digital microscopy has become an essential tool in pathological research over the past few decades. Initially, cameras mounted on microscopes enabled the generation of standard digital images, but the invention of the whole slide scanner over 20 years ago, glass slides can now be scanned to create a whole slide image (WSI) enabling the tissue to be viewed at multiple magnifications (see Fig. 2). The generation of digital images of histological tissue has allowed the creation and application of image analysis (IA) algorithms. More recently still, artificial intelligence (AI) models have been applied to these images and yielded promising results^8–12^.

There is a clear need to improve our current subjective histopathological assessment of cutaneous melanoma, which may be achieved by the implementation of image analysis algorithms. There have been several studies of IA applied to melanoma digital slides, all of which have reported variable methodologies and performances. Prior to any algorithm being adopted into clinical workflows, extensive clinical validation is required, the first step of which would be to provide sufficient evidence to indicate that a model is likely to meet end user requirements. This represents the rationale for this review which summarises the existing evidence and evaluates the performance of these algorithms.

## Materials and methods

This systematic review and meta-analysis was written and performed in accordance with our protocol (PROSPERO ID 336,714) and the Preferred Reporting Items for Systematic Reviews and Meta-Analyses-Diagnostic Test Accuracy (PRISMA-DTA) Statement^13^.

### Participants and studies

We included studies of any design that reported accuracy outcomes of IA applied to histological images of cutaneous melanoma.

### Target condition

The target condition was cutaneous melanoma.

### Index test

The index test was any form of automated IA. This includes more conventional IA techniques as well as neural networks. Manual annotation of histological images was not included.

### Reference standard

The reference standard was any form of histopathological assessment of the haematoxylin and eosin (H&E) histological image and/or immunohistochemical staining.

### Search strategy

PubMed and Embase were searched from inception to 11th March 2022, restricted to English language (for full search strategy see Appendix 1). Citation checking was also conducted.

### Study selection

All citations were independently deduplicated and screened by ELC and RGW. Where possible, the full texts of potentially eligible articles were obtained and independently assessed by the same two individuals with disagreements resolved by discussion. We included abstracts as well as full texts.

### Data extraction

Data were extracted concerning study demographics; type of IA; type of reference standard; conditions included and test statistics to construct 2 × 2 tables of the number of true-positives (TP), false-positives (FP), false-negatives (FN) and true-negatives (TN).

### Methodologic quality assessment

The QUADAS-AI tool was in development at the time of carrying out this work and therefore a tailored version of the Quality Assessment of Diagnostic Accuracy Studies QUADAS-2 was created (per a recent important article in Nature^14^) and used to appraise the risk of bias and applicability of the included studies (Appendix 2).

Assessment of risk of bias for patient selection included whether there was one WSI per patient and if they were contained within one set, since studies that involved multiple WSIs per patient with cases from the same patient spread across the training and test sets result in an overestimation of the model’s performance. Risk of bias with regards to the index test included details of the presence of a separate (ideally external) test set and whether all the cases were included in the analysis. Studies that do not use a separate test set are also at risk of overestimating a model’s performance. Bias of the reference standard included whether the reference standard results were interpreted without knowledge of the index test, alongside the ability of the reference standard to correctly identify melanoma. If the reference standard is interpreted with knowledge of the index test, then this may bias the reference standard to mirror the index test results, again overestimating performance. Finally, studies including cases with a time interval of more than 10 years between the diagnosis of the reference standard and the digital image creation indicated a high risk of bias for flow and timing, since diagnostic criteria and terminology changes with time and glass slides fade introducing risk that they may not clearly depict the pathology and underestimate the model’s performance.

Applicability assessment involved whether the case selection, index test or reference standard matched the review question.

This data was summarised using the Risk-of-bias VISualisation (robvis) tool^15^.

### Statistical analysis

The MetaDTA: Diagnostic Test Accuracy Meta-Analysis v2.01 shinnyapp^16,17^ was used to generate summary sensitivities, specificities, forest plots and summary receiver operating characteristic (SROC) plots using a bivariate random-effects model. A sensitivity analysis was performed including only those studies generating IA models concerned with melanoma tumour detection. A flow-diagram was generated using the PRISMA2020 tool^18^. Publication bias was not assessed because the determinants are not well understood for diagnostic accuracy reviews^19^ and the Deeks test has low power in the presence of substantial heterogeneity^20^. The significance level was set at 5%. Confidence intervals were generated to the 95% level.

## Results

### Study selection

**Figure 3:**
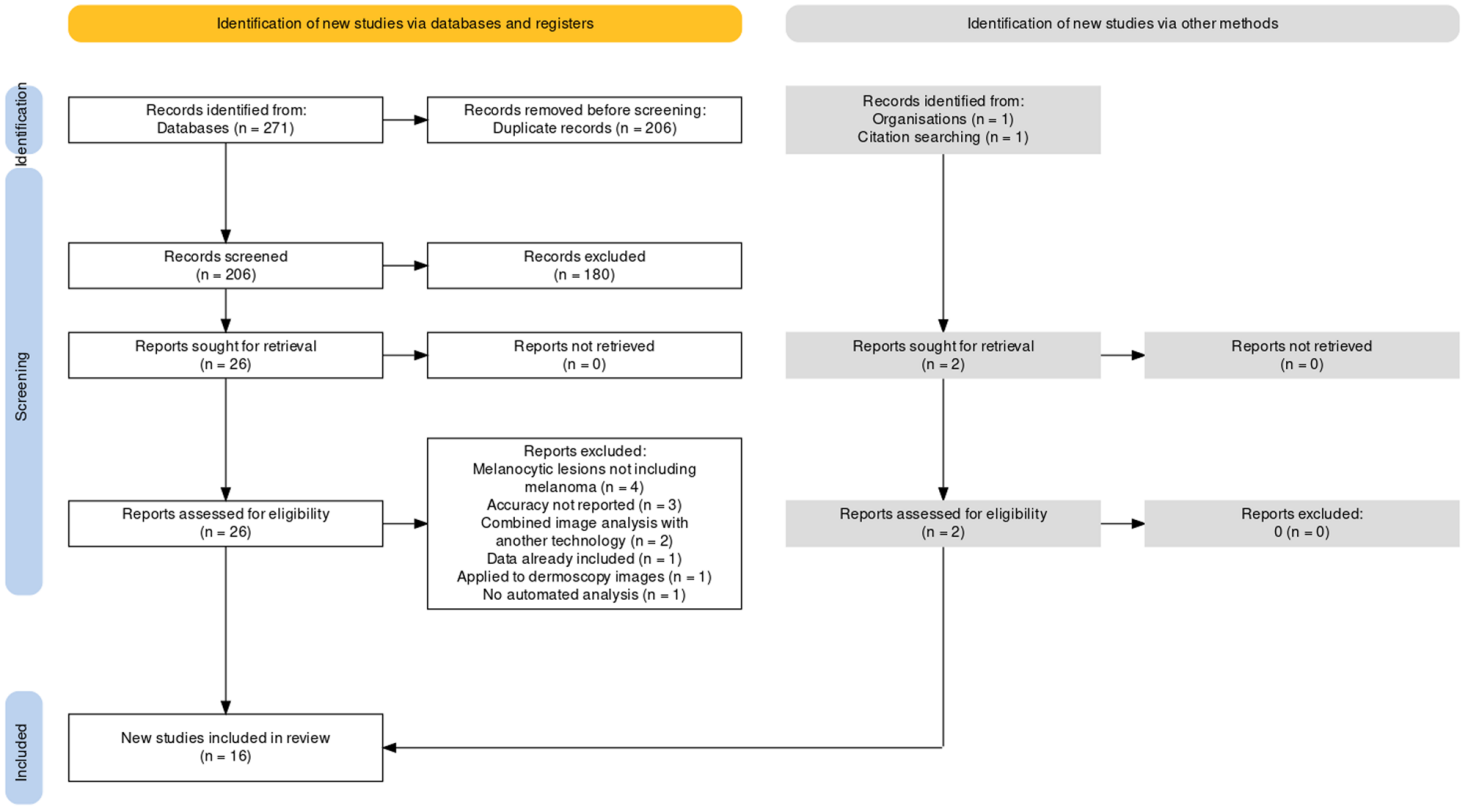
Study flow diagram generated using PRISMA2020 available at: https://estech.shinyapps.io/prisma.

Ultimately, sixteen studies were included (Fig. 3).

### study characteristics

Study characteristics are presented in Supplementary Table 1. Studies originated from the UK^21^, Germany^11,22^, France^23^, Italy^12^, Sweden^24^, USA^10^, Canada^25–28^, Japan^29^ and China^27,30–33^^(p202)^ and were performed between 2012 and 2021. Studies varied in size with a median sample size of 100 specimens or slides (interquartile range [IQR] 66–583.5; range 1–1079). Overall, 4,888 specimens were included, of which at least 2,715 were melanoma specimens. The diagnostic entities within the datasets varied between studies, with some only containing melanoma deposits^12,21,24–26,33^ and others containing more than one pathology^10,11,22,27–32^.

There was between-study variation in terms of intended use of the IA. Most studies focused on a binary classification task, with some focussing on detection and localisation of melanoma deposits in WSIs containing melanoma (melanoma versus not melanoma)^12,25,26,32^ and others performing diagnostic classifications including melanomas versus naevi^11,22,31^ and primary melanoma versus metastatic melanoma^33^. Five studies addressed more complex classifications into three or more diagnostics entities^10,23,28–30^. One study^24^ did not focus on a classification task, but instead studied automation of the proliferation index in melanoma.

There was some degree of variation in the IT used. Most employed the use of a convolutional neural networks (CNN), with the architecture differing considerably^10–12,22,23,26,29–31,33^. Studies using CNNs were more recently conducted. Two of the earliest studies employed the use of a support vector machine (SVM)^24,27^. Two studies used a combination of a CNN and SVM as their index test^11,32^. A further study used more basic image processing and adaptive thresholding method^29^.

There was heterogeneity in the reference standard. In most studies pathologists provided diagnostic labels^10–12,22,31^, categorised specimens by histological features^23^, carried out manual annotation^21,25,28,30^ or interpreted immunohistochemical staining^23,24,26^. Some studies used a combination of these approaches^23,32^. Two studies did not detail the reference standard used^29,33^.

There was variation in the reported units for performance analysis. Some studies reported pixel-based outcomes^25,26^ or cell-level outcomes^24,27,28^, whereas others focussed on patch-level^12,22,31,32^ or slide-level classifications^10,11,30,32^. Three studies did not report on their unit for analysis^23,29,33^, whilst one appeared to be at the WSI-level^23^.

### Risk of bias and applicability concerns

**Figure 4:**
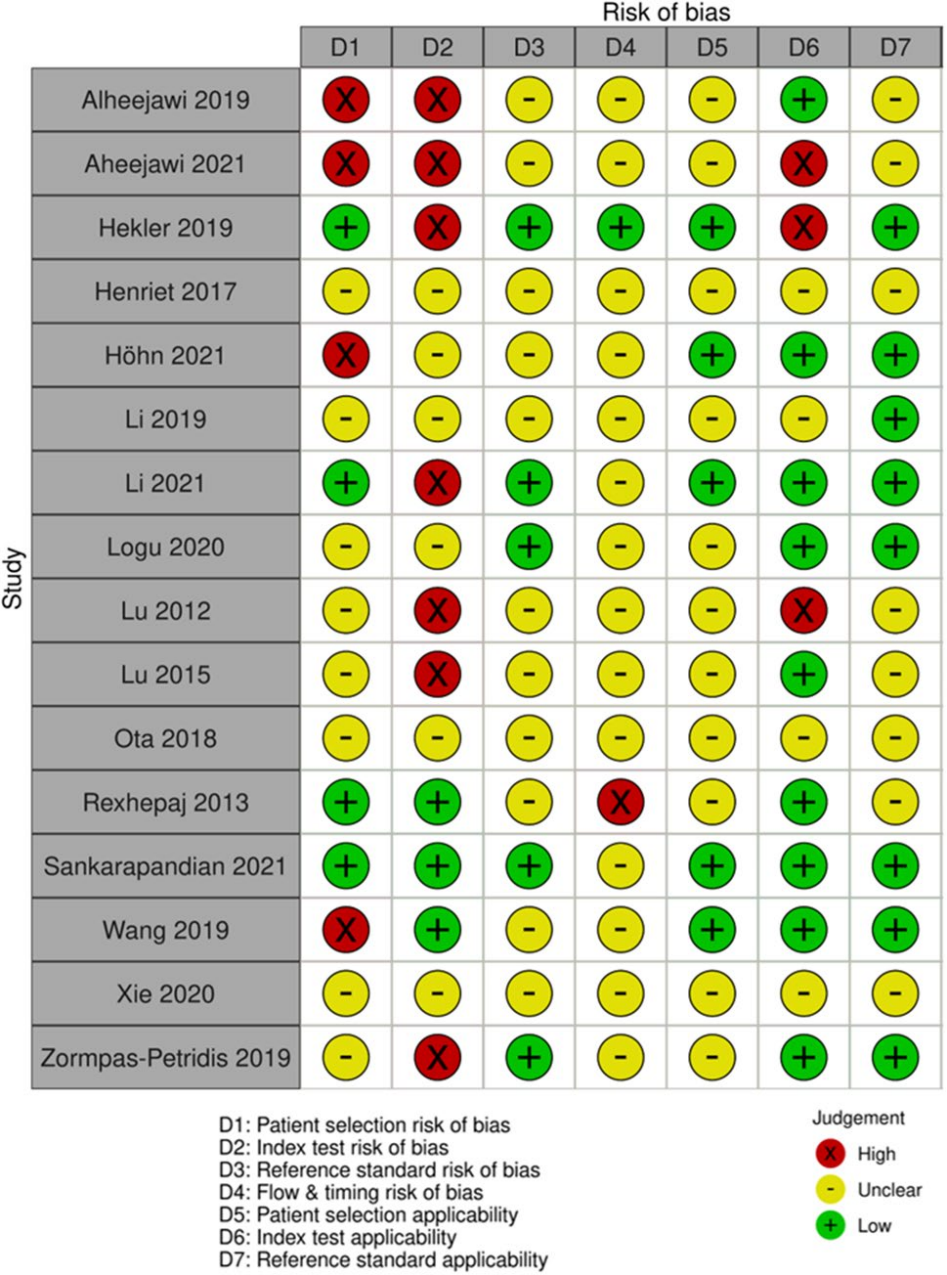
QUADAS-2 summary diagram assessing risk of bias and applicability in the included studies. For more information on how the judgements were made, see Appendix S2.

The risk of bias and applicability assessment are summarised in Fig. 4. Twelve studies were at risk of selection bias^10,11,22–24,26–31,33^, of which, four studies were at high risk since more than one histological image was included per patient and were spread across the training/ test sets^10,26,27,33^. The remaining studies were at unclear risk of patient selection bias^12,21,23,27–29,31,33^. Thirteen studies were at risk of bias from the index test^11,12,21–23,25–31,33^; seven studies were at high risk either due to the index test not being tested on an external test set (i.e., a source separate to those used for training/ validation)^21,22,25,27,30^, or not reporting results from a separate test set^28^, or the test set being derived from the same histological slide as the training and validation sets^26^. Six studies were at unclear risk of bias from the index test^11,12,23,29,31,33^. Eleven studies were at unclear risk of bias from the reference standard^11,23–29,31,31–33^ because it was not clear if the reference standard results were interpreted without knowledge of the IA or if the reference standard was likely to correctly classify the target condition. No studies were at high risk. Fifteen studies were at risk of bias due to the flow and timing^10–12,21,23,25–33^; one study was at high risk of bias since the reference standard was determined over 10 years prior to the index test being conducted^24^. The remaining fourteen studies were at unclear risk of bias since the timings for the determination of the reference standard and index test were not reported^10–12,21,23,25–33^.

Twelve studies were of unclear concern regarding the applicability of patient selection^12,21,23–29,31,33^, due to it not being stated if the cases were purposively selected. There were applicability concerns for seven studies regarding the index test; three studies were of high concern^22,26,28^ and four studies^23,29,31,33^ were of unclear concern that the index test^22,23,26,28,29,31,33^ or its conduct or interpretation differed from the review question. Eight studies^23–29,33^ had unclear concerns for applicability of the reference standard because it wasn’t clear if the individual determining the reference standard was suitably qualified or there were unclear criteria for diagnosis.

### Synthesis of results

Of the sixteen studies included in this systematic review^10–12,21–33^, six studies had data that could be meta-analysed^11,12,21,22,32,33^.﻿ The extracted data from five of these studies were from published work^12,21,22,32,33^﻿ and additional data from one study was provided by the authors^11^. Over these 5 studies, 1,935 specimens were included, of which at least 1,088 were melanoma specimens. The true-positive, false-positive, false-negative and true-negative rates can be seen in Supplementary Table 2.

**Figure 5:**
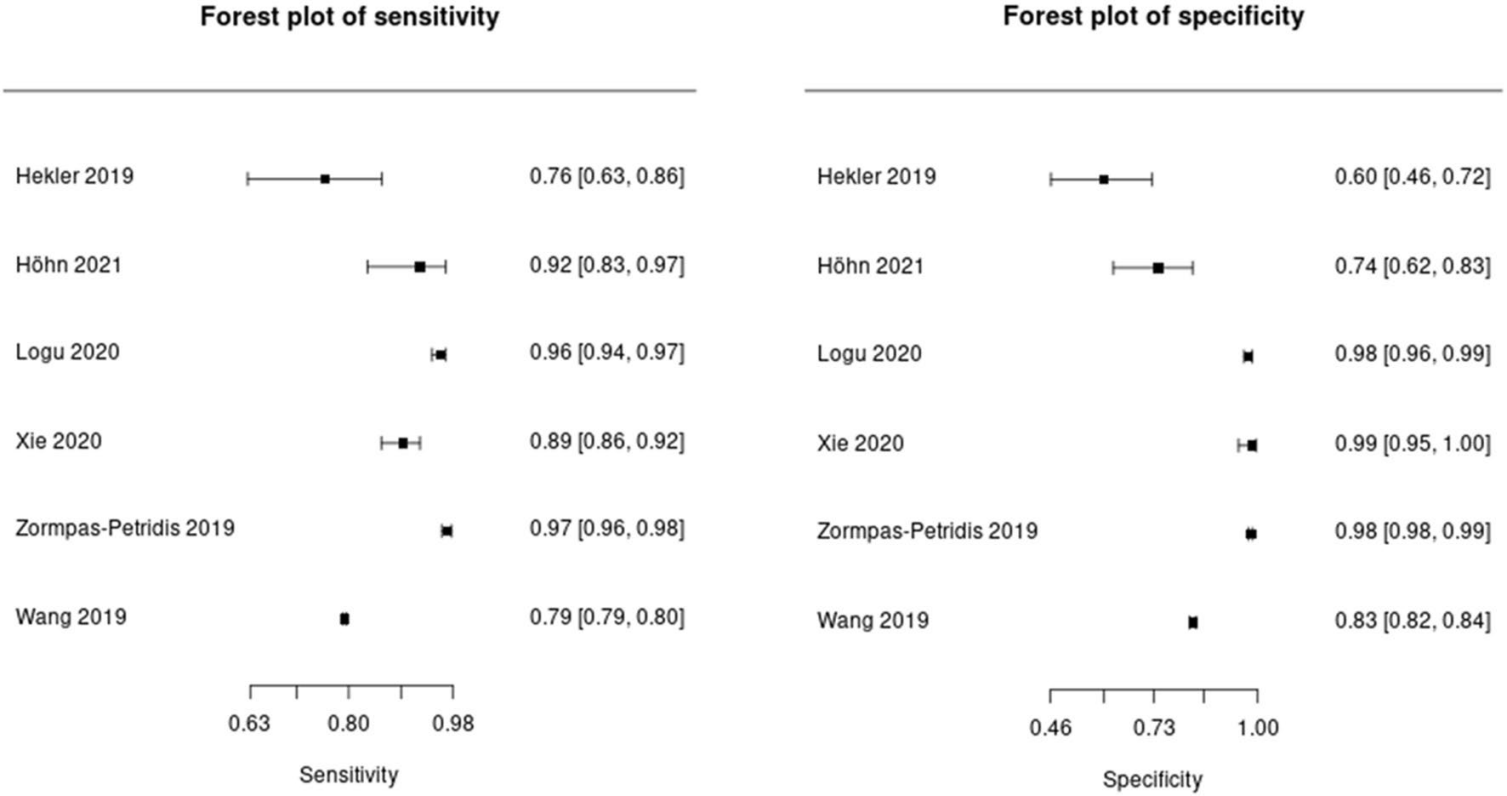
Forest plots of the sensitivity and specificity of image analysis applied to melanoma whole slide images.

**Figure 6:**
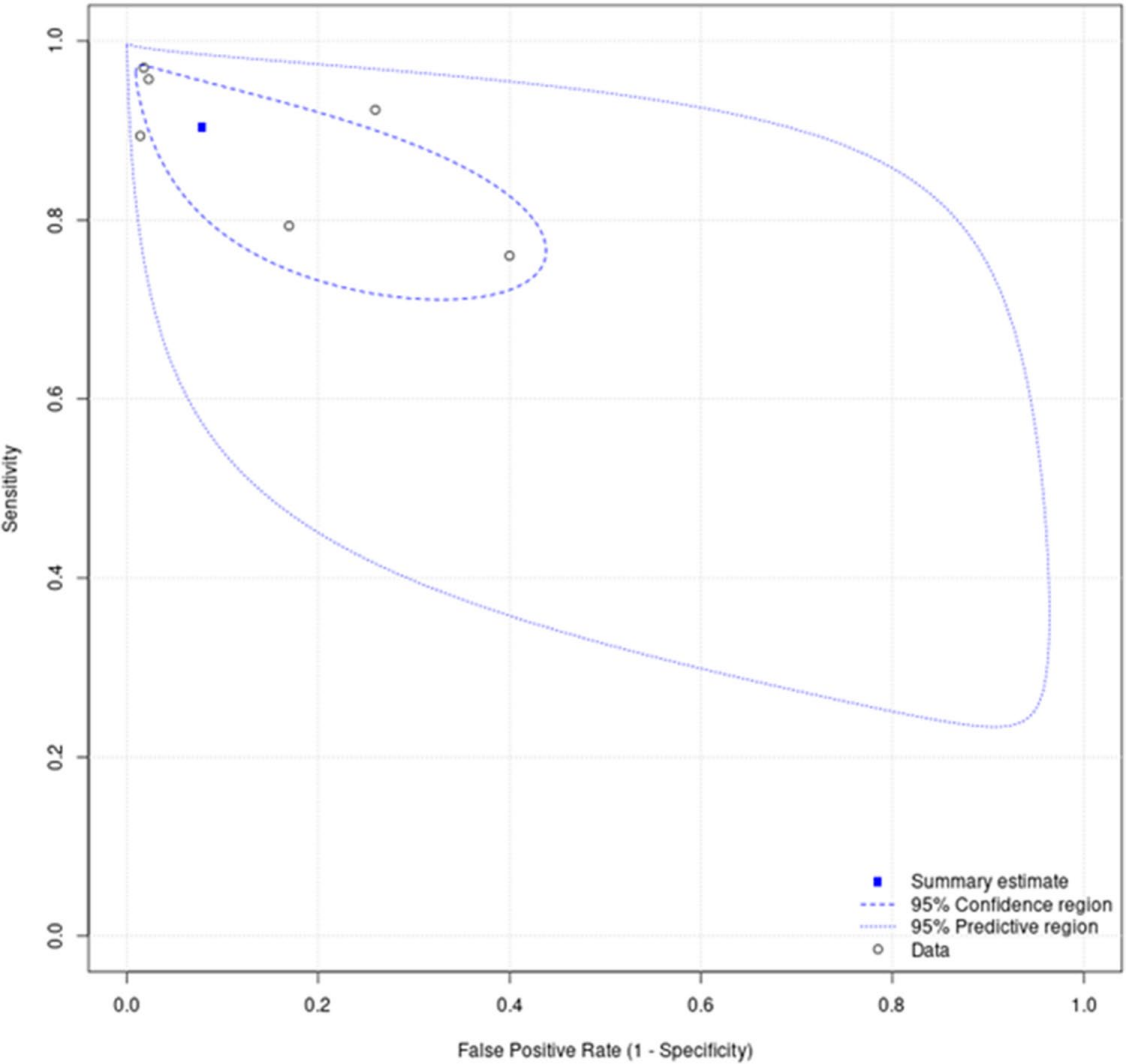
Summary receiver operating characteristic plot of image analysis applied to melanoma whole slide images. The confidence region are the 95% confidence intervals around the summary estimate. The predictive region also captures between study statistical heterogeneity, so depicts the region in which we have 95% confidence that the true sensitivity and specificity of a future study should lie. The predictive region encompasses the possibility that the index test may be worse than chance.

Figure 5 shows forest plots of the sensitivity and specificity of any form of IA applied to cutaneous melanoma histological images. The mean sensitivity was 90% (CI 82%, 95%) and mean specificity was 92% (CI 79%, 97%), as shown in Fig. 6. For the studies which could not be included in the meta-analysis due to deficiencies in reporting, the performance metrics are summarised in Supplementary Table 3.

#### Sensitivity analysis

A sensitivity analysis was performed using the 5 studies concerned with tumour detection^11,12,22,32,33^﻿. In total there were 1,853 specimens, of which at least 1,088 were melanoma specimens. The mean sensitivity of IA for cutaneous melanoma tumour detection was 88% (CI 79%, 93%) and a mean specificity of 90% (CI 71%, 97%).

## Discussion

For all tasks, IA applied to cutaneous melanoma histological images has a high sensitivity and specificity (Fig. 6). When including only those studies concerned with tumour detection, the results were similar. The performance of the models not included in the meta-analysis were also favourable (Supplementary Table 3).

As shown in Fig. 5, three^12,21,33^ of the six meta-analysed studies reported very high sensitivities and specificities, whereas the other three^11,22﻿,32^ were more modest. These three studies^11,22,32^﻿ applied the IA to a reasonably sized separate test dataset containing more than one diagnostic entity. By contrast, the other three studies^12,21,33^ contained less varied datasets containing only melanoma specimens, which may explain the more accurate results.

Across the 16 studies included in this review^10–12,21–33^, there was no clear association between the type of index test or reference standard and the reported performance. Surprisingly, an increase in the number of data sources did not appear to temper performance; two studies^10,12^ contained data from three sources but reported highly accurate results.

Given the exploratory nature of this work, there is currently no consensus regarding whether a more sensitive or specific test is preferable. A more sensitive test would result in fewer false negatives, which in the context of melanoma detection is likely to be of greater utility as missing a melanoma would not be acceptable, particularly as these tests are likely to be used as a screening or triage tool prior to pathologist assessment.

The studies that could not be included in the meta-analysis due to the lack of raw data or data appropriate for back calculation, reported alternative performance metrics including accuracy^25,26,30,31^, dice co-efficient^26^, area under the curve (AUC)^10,24,30^, F-score^24,29,31^, precision^24^, recall^24^, percentage correctly classified^23^, positive prediction rate^28^, under-segmentation rate^28^. The unit of analysis was also variable for the same reasons and included classifications at a pixel-level^25,26^, cell-level^24,27,28^, patch-level^12,22,31,32^ ﻿and slide-level^10,11,30,32^﻿. This variety in reported unit of analysis and performance metrics presents challenges for interpretation and data amalgamation, but it is expected given the wide range of model tasks included in this review. It is essential that the unit of analysis is appropriate for the task to prevent inaccurate performance estimation, as detailed in a seminal paper on the subject^34^. However, regardless of the unit of analysis or performance metrics presented, we urge authors to report their raw data in a confusion matrix (containing the TP, FP, TN, FN counts) for classification-based tasks as per existing guidelines^35^.

The clinical utility of the studies presenting results at the slide-level was clear; to assist with specimen triage^10,11,22,23,30,31,33^. However, many studies which detected melanoma at a cell, pixel or patch-level did not address the clinical utility of their models^21,25–29^, when these models are suited to prognostic biomarker generation. This may be due to difficulties acquiring the necessary data, but we would recommend that future studies detecting melanoma at a cell, pixel or patch-level, focus on how these models could be applied to predict patient outcome.

Our review had limitations. While 16 studies were included in the review, data extraction was only possible for six of the studies owing to deficient reporting^11,12,21,22,32,33^.﻿ There was concern for risk of bias and applicability in all included studies, although reporting standards and methodological rigor did appear to improve with time. This variation in methodological rigor and reporting standards is likely due to a lack of reporting guidelines, although these are currently under development^14^. Additionally, our risk of bias and applicability assessments may be suboptimal since the QUADAS-AI tool was still in development at the time of completion of this work. Future reviewers should deploy this AI-specific tool.

## Conclusion

Based on limited and heterogenous data, IA offers high accuracy when applied to melanoma histological images. The focus of work to date has been on developing the technology in this field, which has accelerated over the past decade. Going forwards, future work should address the clinical application of such models and evaluate their use as a screening/ triage tool or for prognostic/ predictive biomarker generation. The quality of existing studies is variable but is improving with time—it is important that authors report their data according to AI-specific guidelines^14^ once they are published.

## Supplementary Information

Supplementary Information 1.

Supplementary Information 2.

Supplementary Information 3.

Supplementary Information 4.

Supplementary Information 5.

Supplementary Information 6.

## Data Availability

Data used to derive the results presented in this paper are available in the supplementary material.
