# Supplementary material for: Image analysis of cutaneous melanoma histology: a systematic review and meta-analysis
Source: Sci Rep. 2023 Mar 23;13:4774. doi: 10.1038/s41598-023-31526-7 (PMC10036523; doi:10.1038/s41598-023-31526-7)
Supplement: Supplementary file 1 — Supplementary Information 1. [file 41598_2023_31526_MOESM1_ESM.docx]

# Appendix S1

**Melanoma Image Analysis DTA Review Search Strategy**

Pubmed (MEDLINE 1966 onwards)

1. (melano*).ti,ab
2. (n?ev*).ti,ab
3. (digital pathol*).ti,ab
4. (whole slide image).ti,ab
5. (histopathol*).ti,ab
6. (image analysis).ti,ab
7. (support vector machine).ti,ab
8. (random forest).ti,ab
9. (artificial intelligence).ti,ab
10. (machine learning).ti,ab
11. (1 OR 2)
12. (3 OR 4 OR 5)
13. (6 OR 7 OR 8 OR 9 OR 10)
14. (11 AND 12 AND 13)

EMBASE 1947 onwards

1. (melano*).ti,ab
2. (n?ev*).ti,ab
3. (digital pathol*).ti,ab
4. (whole slide image).ti,ab
5. (histopathol*).ti,ab
6. (image analysis).ti,ab
7. (support vector machine).ti,ab
8. (random forest).ti,ab
9. (artificial intelligence).ti,ab
10. (machine learning).ti,ab
11. (1 OR 2)
12. (3 OR 4 OR 5)
13. (6 OR 7 OR 8 OR 9 OR 10)
14. (11 AND 12 AND 13)
