# Supplementary material for: Image analysis of cutaneous melanoma histology: a systematic review and meta-analysis
Source: Sci Rep. 2023 Mar 23;13:4774. doi: 10.1038/s41598-023-31526-7 (PMC10036523; doi:10.1038/s41598-023-31526-7)
Supplement: Supplementary file 2 — Supplementary Information 2. [file 41598_2023_31526_MOESM2_ESM.docx]

# Appendix S2

**QUADAS-2: Image analysis applied to melanoma whole slide images**

**Study ID:**

Cases, setting, intended use of index test, presentation:

Index test (s):

Reference standard and target condition:

1. **Case Selection**

***Risk of bias:***

Describe the methods of case selection:

*Was there one whole slide image per patient? Yes/No/Unclear*

*If no, does the study specify that the images from the same patient kept to within the same set (i.e. not spread across the training and test sets)? Yes/No*

*Did the study avoid inappropriate exclusions? Yes/No/Unclear*

**Could the selection of cases introduced bias? LOW/HIGH/UNCLEAR**

Code low risk if all the answers to signalling questions were ‘yes’

Code high risk if any of the answers to signalling questions were ‘no’

Code as unclear if either answer to signalling questions was ‘unclear’

***Applicability:***

Describe included cases:

**Is there concern that the included cases do not match the review question? LOW/HIGH/UNCLEAR**

Code as low risk if all cases were not purposively selected

Code as high risk if all cases were purposively selected

Code as unclear concern if it is not clear how cases were selected

1. **Index test**

***Risk of bias:***

Describe the index test and how it was conducted and interpreted

*Were the reported results from a separate test set? Yes/No/Unclear*

*Was the index test tested on an external test set (i.e. one from a separate source to those used for training/ validation)? Yes/No/Unclear*

*Did all the cases receive the same image analysis? Yes/No/Unclear*

*Were all test cases included in the analysis? Yes/No/Unclear*

**Could the conduct or interpretation of the index test introduced bias? LOW/HIGH/UNCLEAR**

Code low risk if all the answers to signalling questions were ‘yes’

Code high risk if any of the answers to signalling questions were ‘no’

Code as unclear if either answer to signalling questions was ‘unclear’

***Applicability:***

**In this review, since the Is there concern that the index test, its conduct or interpretation differ from the review question? LOW/HIGH/UNCLEAR**

Code as low risk if there is no concern that the index test, its conduct or interpretation differ from the review question

Code as high risk if there is concern that the index test, its conduct or interpretation differ from the review question

Code as unclear concern if it is not clear if the index test, its conduct or interpretation differ from the review question

1. **Reference standard**

***Risk of bias:***

Describe the reference standard test and how it was conducted and interpreted

*Were the reference standard results interpreted without knowledge of the index test results? Yes/No/Unclear*

*Is the reference standard likely to correctly classify the target condition? Yes/No/Unclear*

**Could the conduct or interpretation of the reference standard introduced bias? LOW/HIGH/UNCLEAR**

Code low risk if both the answers to signalling questions were ‘yes’

Code high risk if any of the answers to signalling questions were ‘no’

Code as unclear if either answer to signalling questions was ‘unclear’

***Applicability:***

**Is there concern that the target condition as defined by the reference standard does not match the review question? LOW/HIGH/UNCLEAR**

Code as low risk if the criteria for diagnosis was clearly defined and the target condition was diagnosed by a suitably qualified individual

Code as high risk if the criteria for diagnosis was not clearly defined or if the target condition was not diagnosed by a suitably qualified individual

Code as unclear concern if the criteria for diagnosis of melanoma was unclear/ incompletely described, or if it is not clear who diagnosed the target condition

1. **Flow and timing**

***Risk of bias:***

Describe the time interval between index test(s) and the reference standard

*Was the time interval between diagnosis of the reference standard and the scanning of the glass slides to create whole slide images >10 years? Yes/No/Unclear*

**Could case flow have introduced bias? LOW/HIGH/UNCLEAR**

Code low risk if all the answers to signalling questions were ‘yes’

Code high risk if any of the answers to signalling questions were ‘no’

Code as unclear if either answer to signalling questions was ‘unclear’
