# Supplementary material for: Image analysis of cutaneous melanoma histology: a systematic review and meta-analysis
Source: Sci Rep. 2023 Mar 23;13:4774. doi: 10.1038/s41598-023-31526-7 (PMC10036523; doi:10.1038/s41598-023-31526-7)
Supplement: Supplementary file 3 — Supplementary Information 3. [file 41598_2023_31526_MOESM3_ESM.docx]

| **Lead author, publication year & reference** | **Available data** | **Index test** | **Intended use** | **Reference standard** | **Sample size across all sets** | **Dataset source (availability)** | **Separate test set?** | **Unit for analysis** | **Funding source(s)** |
| --- | --- | --- | --- | --- | --- | --- | --- | --- | --- |
| Alheejaw 2019^25^ | Full text | SVM | Melanoma deposit detection & proliferation index in lymph nodes, stained using H&E & IHC | Manual annotation by doctors | 39 WSIs | Cross Cancer Institute, University of Alberta (closed) | Yes (internal source) | Pixel-level | Natural Sciences and Engineering Research Council of Canada (NSERC) |
| Alheejawi 2021^26^ | Full text | CNN | Detect H&E melanoma regions | MART1 staining of consecutive section | 100 960x960 pixel images | Cross Cancer Institute, University of Alberta (closed) | Yes (internal source) | Pixel-level | Natural Sciences and Engineering Research Council of Canada (NSERC), Ministry of Higher Education and Scientific Research (MOHESR), Iraq and Imam Ja’afar Al Sadiq University, Iraq |
| Hekler 2019^22^ | Full text | CNN | Classify H&E cropped images into melanoma & benign naevi | 11 histopathologist classification | 695 cropped images | Dr Dieter Krahl institute, Heidelberg (closed) | Yes (internal source) | Patch-level | None declared |
| Henriet 2017^23^ | Abstract | CNN | Classification of WSIs into spitz naevi, atypical spitz tumour & melanoma | 18 morphological characteristics & 4 IHC markers determined by unknown individual | 66 cases | Unknown | No (cross-over validation) | Unknown but likely Slide-level | N/A |
| Hohn 2021^11^ | Full text & additional data | CNN | Classify H&E WSIs into melanoma & benign naevus | Classification by two experienced pathologists | 431 WSIs | Two ‘laboratories' (closed) | Yes | Slide-level | Federal Ministry of  Health, Berlin, Germany and Tumor Behavior Prediction Initiative |
| Li 2019^31^ | Abstract | CNN | Classify H&E stained 224x224 pixel patches into melanoma & benign naevi | 10 certified dermatopathologist classification | 2186 WSIs | Xiangya Hospital Medical Association (closed)  TCGA (open) | Unknown | Patch-level | N/A |
| Li 2021^30^ | Full text | CNN | Lesion detection & distinguish between melanoma, intradermal naevi, junctional naevi, compound naevi & normal tissue | Manual annotation by a pathologist | 701 WSIs | Central South University Xiangya Hospital (closed)  TCGA (open) | Yes (internal source) | Slide-level | Not stated |
| Logu 2020^12^ | Full text | CNN | Segment H&E WSIs into melanoma & healthy tissue | Two expert dermatopathologist classification | 100 WSIs | University of Florence (closed)  University Hospital of Siena (closed)  Institute of Biomolecular Chemistry, National research Council (closed) | Yes | Patch-level | Associazione Italiana per la Ricerca  sul Cancro (AIRC) |
| Lu 2012^28^ | Full text | Basic image processing & adaptive thresholding | Detect nuclei in normal skin, melanocytic naevi & melanomas | Manual annotation of nuclei by unknown individual | 30 512x512 pixel images | Unknown | No | Cell-level | Not stated |
| Lu 2015^27^ | Full text | SVM | Categorise tissue into melanoma, naevus or normal skin | Manual annotation by unknown individual | 66 WSIs | Unknown | Yes (internal source) | Cell-level | National Natural Science Foundation of China and Fundamental Research Funds for the Central Universities of China |
| Ota 2018^29^ | Abstract | 2 CNNs | Detect atypical cells, epidermis, dermis & subcutaneous tissue | Unknown but likely manual annotation/ classification by pathologist | 143 WSIs | Unknown | Unknown | Unknown | N/A |
| Rexhepaj 2013^24^ | Full text | SVM | Quantify proliferation index of melanomas | Manual Ki67 assessment | 334 TMAs | Unknown sources of two datasets “discovery and validation cohorts” (closed) | Yes (external source) | Cell-level | EU 7th Framework Programme under the auspices of the Marie Curie Industry-Academia Partnership  and Pathways program, Target-Melanoma and the Knut and Alice Wallenberg Foundation |
| Sankarapandian 2021^10^ | Pre-print full text | CNN | Triaging specimens into basaloid, squamous, melanocytic low risk (MPATH-DX I or II), melanocytic intermediate risk (MPATH-DX III), melanocytic high risk (MPATH-DX IV or V) or other | Consensus classification by up to 5 dermatopathologists | 12,784 WSIs | University of Florida College of Medicine (closed)  Thomas Jefferson University (closed)  Cedas-Sinai Medical Center (closed) | Yes (external source) | Slide-level | Not stated |
| Wang 2019^32^ | Full text | CNN for patch-level classification & random forest for WSI-level classification | Detect melanoma in H&E WSIs of eyelid specimens | Diagnostic consensus of two board-certified pathologists using traditional microscopy of H&E +/- IHC & one independent pathologist review & manual annotation  Binary classification by 7 board-certified pathologists on a 9 WSI subset | 155 WSIs (83,126 patches) | Zhejiang University School of Medicine (closed)  Ninth People’s Hospital of Shanghai (closed) | Yes (external source) | Patch & Slide-level | National Natural Science Foundation of  China |
| Xie 2020^33^ | Abstract | CNN | Classification of primary melanomas & metastatic melanomas | Unknown but likely pathologist diagnosis | 472 WSIs | Xiangya Hospital (closed)  Yale Medical Center (closed) | Unknown | Unknown | N/A |
| Zormpas-Petridis 2019^21^ | Full text | SVM & CNNs | Single-cell classification to detect cancer cells, lymphocytes, stromal cells, epidermal cells | Pathologist manual annotation | 105 WSIs | TCGA (open) | Yes (internal source) | Cell-level | Cancer Research UK  NHS funding to the NIHR Biomedicine  Research Centre and the Clinical Research Facility in Imaging,  The Rosetrees Trust,  Breast Cancer Now &  Children with Cancer  UK |

Supplementary Table 1 - Characteristics of included studies
