# Supplementary material for: Image analysis of cutaneous melanoma histology: a systematic review and meta-analysis
Source: Sci Rep. 2023 Mar 23;13:4774. doi: 10.1038/s41598-023-31526-7 (PMC10036523; doi:10.1038/s41598-023-31526-7)
Supplement: Supplementary file 4 — Supplementary Information 4. [file 41598_2023_31526_MOESM4_ESM.docx]

| **Study** | **True positive count** | **False negative count** | **False positive count** | **True negative count** |
| --- | --- | --- | --- | --- |
| Hekler 2019^22^ | 38 | 12 | 20 | 30 |
| Höhn 2021^11^ | 60 | 5 | 17.4 | 49.6 |
| Logu 2020^12^ | 1075 | 48 | 18 | 772 |
| Xie 2020^33^ | 295 | 35 | 2 | 140 |
| Zormpas-Petridis 2019^21^ | 1482 | 46 | 45 | 2486 |
| Wang 2019^32^ | 60259 | 15690 | 1217 | 5960 |

**Supplementary Table 2 – True positive, false negative, false positive and true negative counts for each study included in the meta-analysis. Please note, the results for Höhn 2021^11^ represent the average count over 5 identically trained models tested on the same data.**
