# Supplementary material for: Image analysis of cutaneous melanoma histology: a systematic review and meta-analysis
Source: Sci Rep. 2023 Mar 23;13:4774. doi: 10.1038/s41598-023-31526-7 (PMC10036523; doi:10.1038/s41598-023-31526-7)
Supplement: Supplementary file 5 — Supplementary Information 5. [file 41598_2023_31526_MOESM5_ESM.docx]

| **Study** | **Reported performance** |
| --- | --- |
| Alheejawi 2019^25^ | 90% accuracy |
| Alheejawi 2021^26^ | 94% accuracy, 85% dice co-efficient |
| Henriet 2017^23^ | 91% percentage correctly classified |
| Li 2019^31^ | 91% accuracy, 87% F-score |
| Li 2021^30^ | 0.971 AUC |
| Lu 2012^28^ | 88.11% sensitivity rate, 80.02% positive prediction rate, 5.34% under-segmentation rate |
| Lu 2015^27^ | ~90% accuracy |
| Ota 2018^29^ | 73% F-score |
| Rexhepaj 2013^24^ | 0.914 TP, 0.105 FP, 0.891 precision, 0.914 recall, 0.913 F-score, 0.964 ROC area |
| Sankarapandian 2021^10^ | 0.95 AUC |

Supplementary Table 3 – Other reported performances

from studies not able to be included in the meta-analysis
